# A multilayered cell envelope of a member of the Chloroflexota offers an anchoring platform for the archaellum

**DOI:** 10.3389/fmicb.2026.1850455

**Published:** 2026-06-17

**Authors:** Marie Joest, Clara L. Mollat, Laura Mutschler, Marta Rodriguez-Franco, Shamphavi Sivabalasarma, Friedel Drepper, Pitter F. Huesgen, Thomas Ott, Sonja-Verena Albers

**Affiliations:** 1 Molecular Biology of Archaea, Faculty of Biology, University of Freiburg, Freiburg, Germany; 2 Spemann Graduate School of Biology and Medicine, University of Freiburg, Freiburg, Germany; 3 Cell Biology, Institute of Biology, Faculty of Biology, University of Freiburg, Freiburg, Germany; 4 Biochemistry and Functional Proteomics, Faculty of Biology, University of Freiburg, Freiburg, Germany; 5 CIBSS – Centre for Integrative Biological Signaling Studies, University of Freiburg, Freiburg, Germany

**Keywords:** Archaea, cell envelope, motility, peptidoglycan, Type IV pilus, pili

## Abstract

In a previous study, we discovered that *Litorilinea aerophila*, a member of the bacterial phylum Chloroflexota, had acquired a *bona fide* archaellum gene cluster through horizontal gene transfer from Archaea, a surprising finding given that the archaellum had long been considered an archaeal-specific motility machinery. Here, we hypothesize that the distinctive multilayered cell envelope of *L. aerophila* provides the structural context that enables the integration and function of the archaellum motility machinery. Using fluorescence microscopy, thin-section electron microscopy, and cryo-electron tomography, we revealed the organisation of the *L. aerophila* envelope and propose a mechanism for how the archaellum can traverse the peptidoglycan of *L. aerophila* by using the Type IV pilus alignment complex proteins PilO and PilN. In addition, we identified two other cell surface appendages: (i) pilus-like structures consistent with Tad pili, and (ii) grappling hook-like structures. Structural analysis of the grappling hook by CryoEM revealed an architecture that possibly plays a role in cell–cell interactions. Together, these findings imply that the evolution of a complex, multilayered cell envelope in Chloroflexota has facilitated the functional adaptation of archaeal surface machineries, allowing these bacteria to exploit the archaellum as a simpler, more energy-efficient motility system than the bacterial flagellum.

## 1 Introduction

Swimming motility enables microorganisms to navigate their environment and has evolved multiple times across the domains of life. While eukaryotes use microtubule based cilia and bacteria flagella derived from type III secretion systems; in Archaea, motility is mediated by the archaellum, a rotary propulsive filament that evolved from the type IV filament (TFF) superfamily, whose ancestral members assemble non-rotating filaments involved in adhesion or surface motility (Albers and Jarrell, 2018; Denise et al., 2019; Beeby et al., 2020).

A central challenge in the evolution of rotary motility is torque generation. Rotation of a filament requires a stator that anchors the motor to a rigid cellular structure; without such anchoring,

torque would cause the motor to rotate within the membrane rather than propel the cell forward. Ancestral TFF systems lack a stator and therefore cannot generate rotation (Denise et al., 2019). The emergence of a stator is thus considered a key evolutionary step in the transition from non-rotating filaments to a rotary motility apparatus.

In the archaellum, stator function is mediated by the proteins ArlG and ArlF, which are conserved components of the archaellum operon and are essential for assembly and rotation (Lassak et al., 2012). Both proteins originated from the filament protein ArlB through gene duplication followed by neofunctionalization (Umrekar et al., 2021).

Although ArlF and ArlG have C-terminal Ig-fold domains like the archaellin ArlB, they do not have a class III signal peptide, which is found in true archaellins and essential for their assembly into the archaellum (Tsai et al., 2020). ArlG undergoes proteolytic N-terminal truncation upon secretion, whereas ArlF is secreted in full length (Tsai et al., 2020). Truncated ArlG assembles into open helical filaments that span the pseudoperiplasm (Banerjee et al., 2015; Umrekar et al., 2021). At the distal end of the ArlG filament, an ArlG–ArlF heterotetramer forms a cap, with ArlF directly interacting with the S-layer (Banerjee et al., 2015; Tsai et al., 2020). Disruption of either the ArlG-ArlF interaction or ArlF-S-layer binding interface abolishes motility, demonstrating that anchoring of the stator to the cell envelope is essential for torque generation (Tsai et al., 2020).

For many years, the archaellum was considered restricted to archaea, in part because its function relies on archaeal envelope features, such as the S-layer, which provides a rigid anchoring site for the stator (Beeby et al., 2020). This view was challenged by the discovery of a complete archaellum gene cluster in members of the bacterial phylum Chloroflexota (Hug et al., 2013; Lim et al., 2023). Some members of the Chloroflexota, including the filamentous *Litorilinea aerophila* acquired a bona fide archaellum gene cluster via horizontal gene transfer from archaea. Swimming motility analysis followed by structural analysis confirmed the presence of a functional archaellum filament. However, the mechanism of anchoring this system to a bacterial cell envelope lacking an archaeal S-layer remained unknown (Sivabalasarma et al., 2025). Because stator anchoring is essential for torque generation, understanding how the archaellum interfaces with the bacterial cell envelope is critical for explaining how rotary motility can be achieved outside of an archaeal cell envelope context.

Several members of the phylum of Chloroflexota, including representatives of the classes Chloroflexi, Anaerolineaea, Caldilineae, and Ktedonobacteria, form multicellular filaments and exhibit a distinctive, atypical cell envelope architecture that may provide alternative anchoring structures for rotary motility systems (Sutcliffe, 2011; Hanada, 2014). The cell envelope of Chloroflexota has been of great interest as, despite Gram-negative staining, they are considered monoderm. Despite the multilayered envelope architecture of some Chloroflexota, there is no clear evidence for a lipid outer membrane (van der Meer et al., 2002; Sutcliffe, 2011). The absence of genes of the beta-barrel assembly machinery (BAM-complex) and liposaccharide biosynthesis pathway, which are encoded in all known diderm phyla, supports the lack of an outer membrane (Sutcliffe, 2010). Instead, it is suggested that the layered structures are composed of polysaccharides or proteins (Sutcliffe, 2011). However, recent cryo-electron tomograms of *Candidatus* Viridilinea mediisalina and *Chloroflexus aggregans*, show a diderm-like envelope, yet they lack diderm signature pathways and genes. This suggests that Chloroflexota might harbor mono- and neoderm architecture that needs to be elucidated (Gaisin et al., 2020; Benyahia et al., 2025). These observations suggest that Chloroflexota may possess mono- or neoderm envelope architectures that differ fundamentally from classical bacterial or archaeal models. Such atypical envelope features raise the possibility that novel stator anchoring strategies may exist in Chloroflexota, potentially enabling the functional integration of an archaeal rotary motility system into a bacterial cell envelope.

Previously, we solved the structure of the first bacterial archaellum isolated from *Litorilinea aerophila*, which belongs to the class Caldilineae within the phylum Chloroflexota (Sivabalasarma et al., 2025). This finding raised a central mechanistic question: how can an archaeal rotary motility system be stably anchored in a bacterial cell envelope that lacks the canonical archaeal S-layer? We hypothesised that the atypical, neoderm-like cell envelope architecture of *L. aerophila* provides alternative anchoring structures capable of replacing the archaeal S-layer, thus fulfilling the stator function required for torque generation. Therefore, we examined the complex cell envelope by using electron and light microscopy, which provided insights into the possible anchoring mechanism of the archaellum. Additionally, we described two other filamentous cell-surface appendages, providing broader insight into *L. aerophila* cell-surface structures. Together, these findings address the question of how the physical requirement for the anchoring of the stator is met in a bacterial cell envelope, enabling swimming motility with an archaellum outside of an archaeal context.

## 2 Results

### 2.1 Filamentous growth and morphology dynamics of *Litorilinea aerophila*

**Figure 1:**
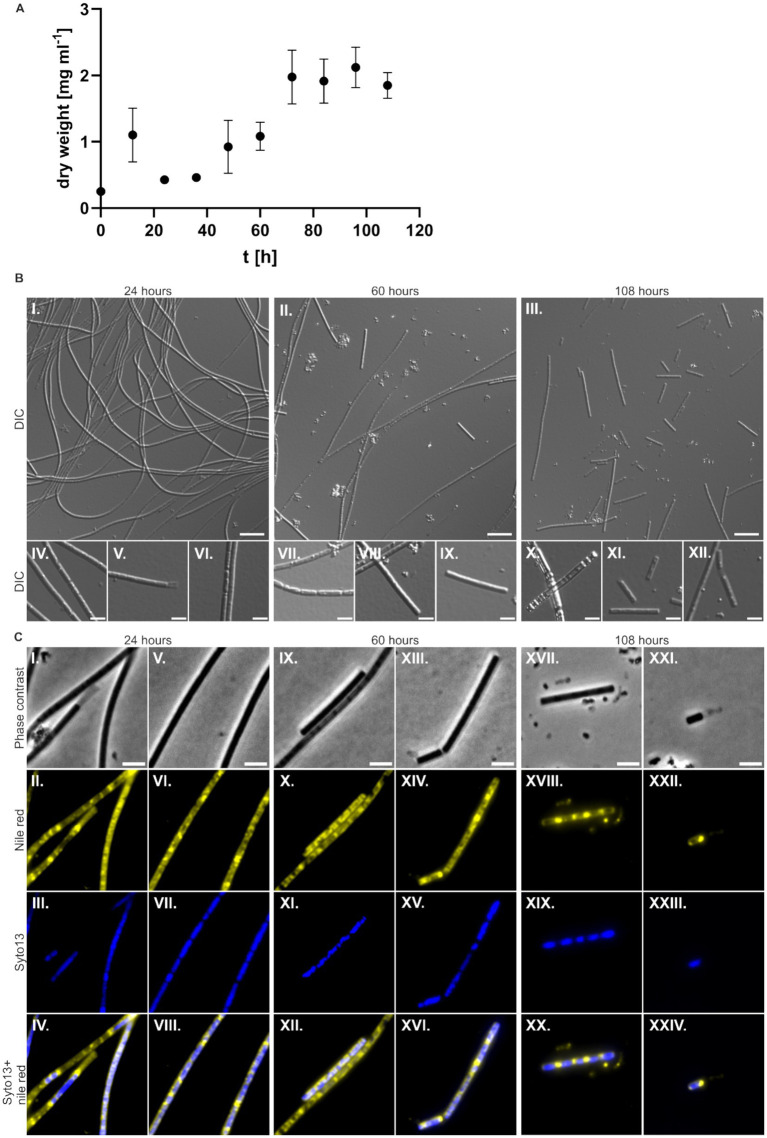
Cell growth and light microscopy images of *L. aerophila* cells cultured in marine broth medium. **(A)** Growth of *L. aerophila* measured as dry weight over 5 days. Each value represents the mean of three independent, biological replicates. Error bars show SEM. **(B)** Differential interference contrast (DIC) light microscopy of *L. aerophila* cells after 24, 60 and 108 h of growth. **(C)** Fluorescent light microscopy of *L. aerophila* cells after 24, 60 and 108 h of growth shown in phase contrast channel. The membrane is stained with Nile red (shown in yellow), DNA is stained with Syto13 (shown in blue). Scale bars of the overview: 10 μm. Scale bar of the zoomed images: 3 μm.

**Figure 2:**
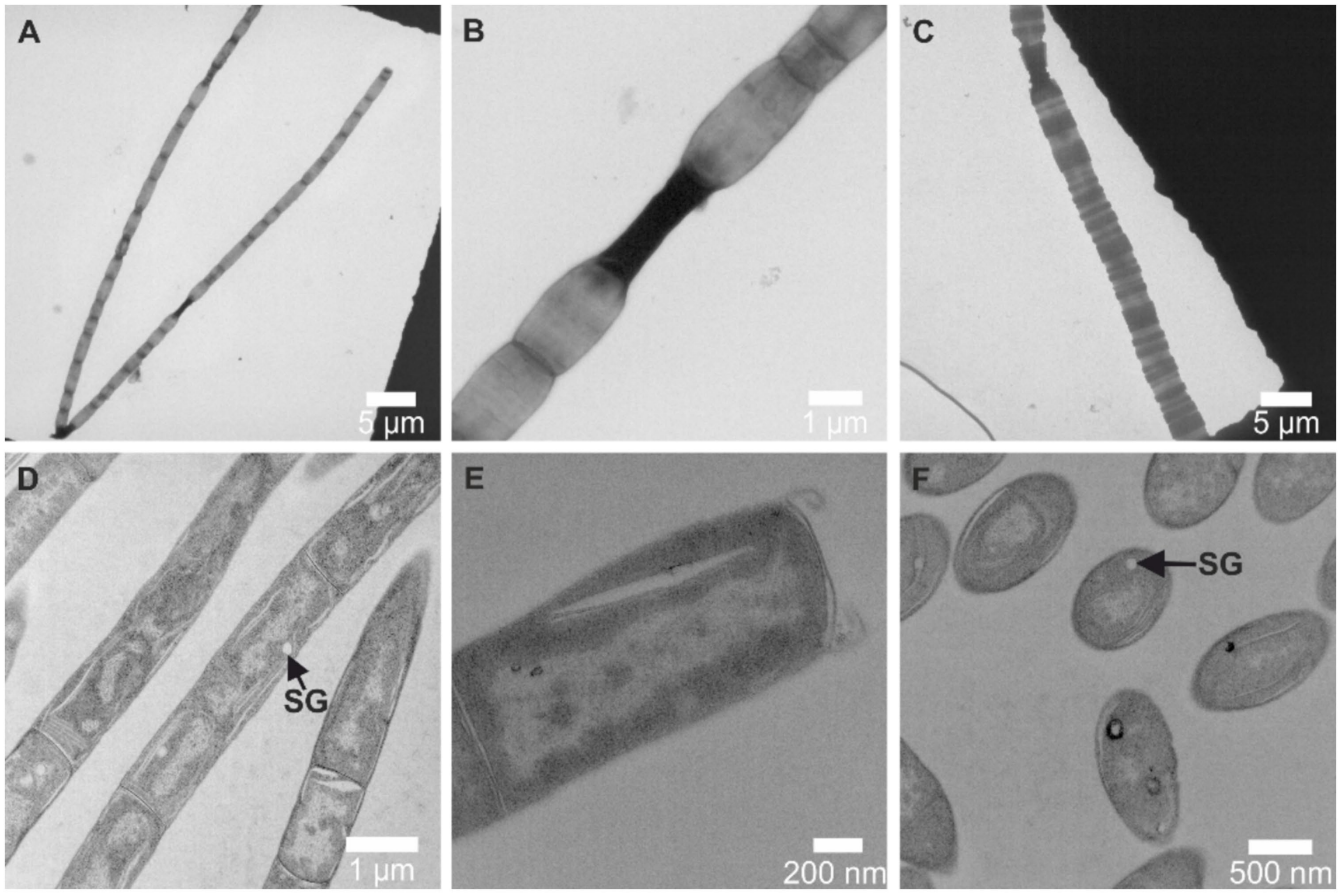
Electron microscopy images of *Litorilinea aerophila*. **(A–C)** Negative-stained transmission electron microscopy images of *L. aerophila* captured in different growth phases: **(A)** Lag phase, **(B)** exponential phase, and **(C)** stationary growth phase. **(D–F)** Ultra-thin sections of *L. aerophila* from the exponential growth phase were prepared for TEM. **(D)** Septal regions, **(E)** cell poles, and **(F)** lateral cross-sections of intracellular organization.

To provide a framework for understanding the archaellum anchoring within the cell envelope, the filamentous growth and spatial organization of *L. aerophila* was analyzed. When cultivated in marine broth medium, *L. aerophila* exhibited a characteristic filamentous morphology previously described by Kale et al. (2013) (Figure 1B I.). Due to extensive clumping and the culture’s filamentous nature, standard optical density measurements were not possible for growth quantification. Instead, dry weight was measured over a period of 5 days, revealing a cell mass doubling time of 19.25 h during the exponential growth phase in marine broth medium (Figure 1A). To monitor morphological changes during growth, cells were analyzed at regular intervals by light and fluorescence microscopy (Figures 1B,C). As cultures reached higher cell densities, sediments from the medium increasingly accumulated and were visible as background material (Figure 1B II., III.). While these sediments were largely negative for Nile red staining compared to the multicellular filaments and membrane blebs, weak Nile red staining was occasionally observed in a subset of sediment particles, suggesting that at least part of these are membrane-derived components (Figure 1C XVII.–XX.). Differential interference contrast microscopy (DIC) revealed irregular spaced membrane indentations within the cell filament, suggestive of multicellular organization (Figure 1B, I., II., IV., VI., VII.) These membrane indentations were confirmed by fluorescence microcopy using nile red as membrane stain (Figure 1C II., VI., X., XIV., XVIII.) and were also observed in negative-stain transmission electron microscopy (Figures 2A,B). Dark constricted regions within the multicellular filament were observed in TEM, however, their biological significance remains unclear (Figure 2B). Diffuse DNA staining between membrane indentations further supported the interpretation of a multicellular organization, indicating the presence of compartmentalized nucleoids within individual filaments (Figure 1C, VI.–XX.). Filament lengths frequently exceeded 100 μm (Figure 1B, I.–III.).

Transmission electron microscopy of thin sections confirmed the presence of membrane septa separating individual compartments within filaments (Figure 2D), consistent with earlier reports in other filamentous Chloroflexota species (Gaisin et al., 2020). Interestingly, polar segments of filaments occasionally lacked a detectable DNA signal by fluorescence microscopy (Figure 1B V., C I., II., Supplementary Figure 1). These segments likely correspond to empty membrane remnants left at filament ends due to breakage events (Supplementary Figure 2), consistent with the absence of phase-dark cytoplasmic content in corresponding light microscopy images and with the presence of membrane-like material at polar segments in electron microscopy images (Figure 2E).

As cultures progressed through exponential and stationary phases, the overall filament length shortened (Figure 1B II., VII.–IX.). Membrane indentations became more regular, and smaller multicellular units were frequently detected (Figure 1B VII., C IX.–XVI.). In the stationary phase (after 108 h), filaments were further reduced in length (Figure 1B III., C XVII.–XXIV.). Additionally, vesicle-like membrane blebs were frequently seen budding from filament ends or indentation sites, indicating active membrane remodeling (Figure 1B XII., C XVIII., XX.).

A particularly striking morphological phenotype was occasionally observed during the stationary phase: filaments exhibiting a highly convoluted, tape-worm-like envelope morphology (Figure 1B X.). The biological function or structural basis of this phenotype remains unclear, but it was also observed in electron micrographs (Figure 2C), suggesting a reproducible, possibly stress-induced structural adaptation of the cell envelope.

Time-lapse microscopy further revealed that filament elongation was occasionally interrupted by cell breakage events, resulting in shortening of multicellular filaments (Supplementary Figure 2, Supplementary Movie 1). These breakage events occurred irregularly over the length of the filament and were sometimes accompanied by membrane bleb formation (Supplementary Figure 2, arrows), suggesting localized envelope remodeling.

### 2.2 The cell envelope of *L. aerophila* exhibits a rigid, complex outer structure that supports archaellum anchoring

**Figure 3:**
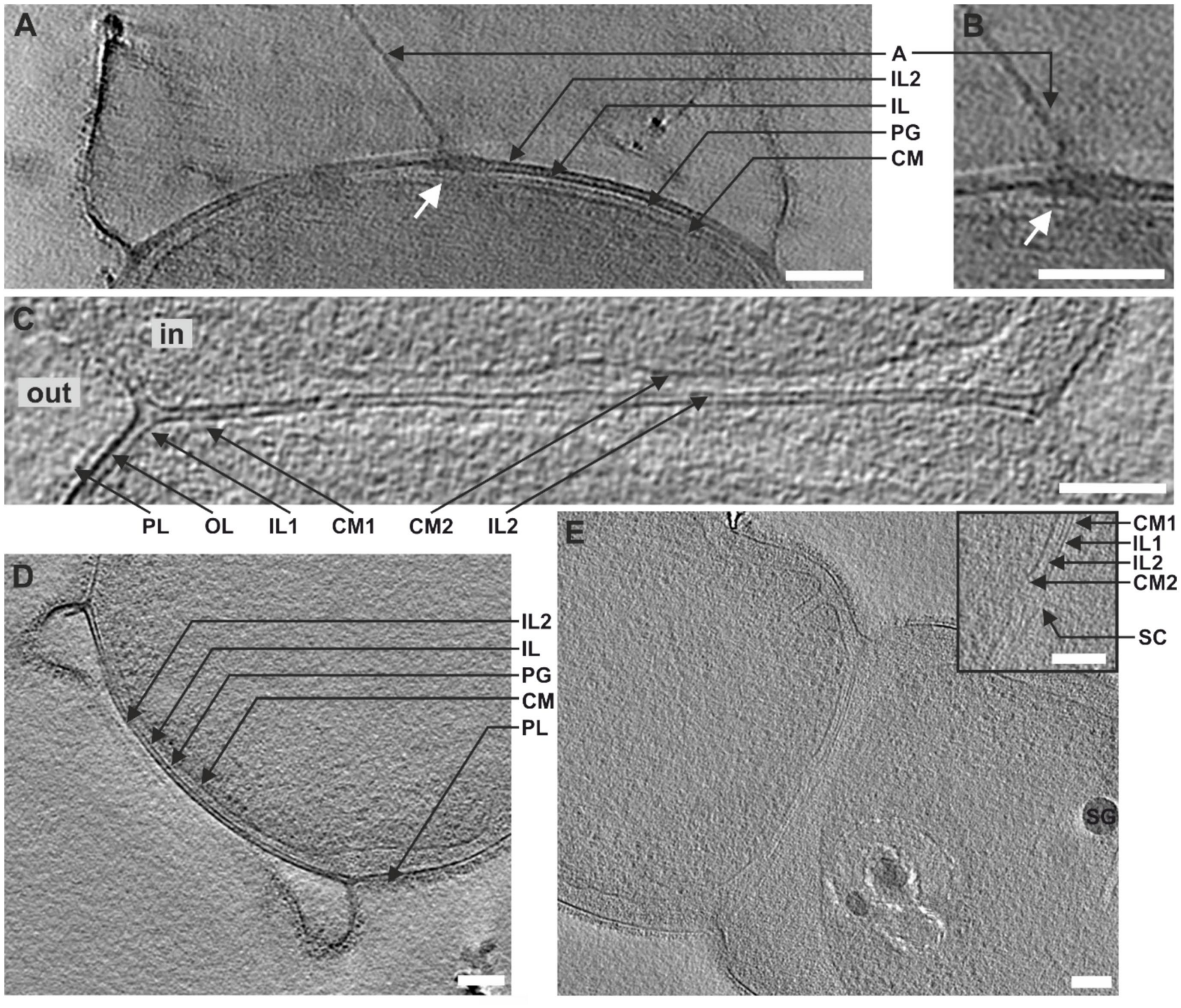
Cryo-electron tomography. **(A)** Cryo-electron tomography of a cell pole of an archaellated cell with a cup-like structure localized between the inner layer and the cytoplasmic membrane (white arrow). **(B)** Close-up of the archaellum anchored to the cell envelope. **(C)** Close-up of a cell-to-cell connection from a cryo-electron tomogram. **(D)** Cryo-electron tomogram of the cell pole. **(E)** Cryo-electron tomogram from *L. aerophila* cells. The inset in the upper right corner shows a magnified view of the septum channel between two adjacent cells. Abbreviations: In, inside of the cell; Out, extracellular space; IL, inner layer; CM, cytoplasmic membrane; OL, outer layer; PL, protein layer; SC, septum channel; A, archaellum; SG, storage granule. Scale bars: 100 nm.

To gain further insight into the ultrastructure of the cell envelope, cryo-electron tomography (cryoET) was performed on vitrified samples of logarithmically growing *L. aerophila* cells, as well as on cells grown on motility plates to induce archaellation. CryoET revealed a multilayered cell envelope (Figure 3). Individual cells within the filament contained a ribosome-filled space enclosed by a cytoplasmic membrane (CM, Figures 3C,E). In addition, cryoET and thin sectioning TEM revealed the spherical, electron-dense structures within the cytoplasm that resemble storage granules as previously reported (Gaisin et al., 2020) (SG, Figures 2D,F, 3E). Each cell was individually enclosed by an additional layer. Because the molecular composition of this layer is unknown, we termed it the inner layer (IL; Figures 3C,E). The entire multicellular filament was further enclosed by a shared outer layer (OL; Figures 3C,E).

This outer layer (OL) was decorated along the lateral side of the filament by a continuous, putative proteinaceous layer (PL, Figures 3C,D), exhibiting a characteristic lattice-like density that surrounded the entire multicellular filament, consistent with the morphology of a surface (S)-layer. Due to the orientation of individual tomographic reconstructions, the PL and OL are visible only over a limited stretch in any single tomogram, but were consistently observed covering the lateral filament surface. Adjacent cells were separated from each other by their respective cytoplasmic membrane and inner layer within the filament (CM1, IL1 and IL2, CM2, Figure 3E). A connection between cell junctions between two cells was found reminiscent of septum channels found in other filamentous Chloroflexota (Gaisin et al., 2020) (SC, Figure 3E). This septum channel (SC) connects the cytoplasmic membranes (CM) of the individual cells (Figure 3E, close-up).

At the polar ends of filaments, a distinct organisation of the cell envelope was observed compared to the lateral cell envelope (Figure 3). Notably, neither the outer layer (OL) nor the proteinaceous layer (PL) was detected at the cell poles, regardless of whether cells were archaellated (Figures 3A,B) or non-archaellated (Figure 3D). This indicates localized remodelling of the cell envelope at the cell pole, independent of the archaellation of the cells. Instead, an additional inner layer was observed at the polar region (IL2, Figures 3A,D), interpreted here as a remnant inner layer of a previously attached cell, consistent with the filament breakage events described above (Supplementary Figure 2). A layer resembling peptidoglycan was resolved at the polar region between the cytoplasmic layer and the inner layer in both archaellated and non-archaellated cells (PG, Figures 3A,B,D). While this layer was not clearly resolvable along the lateral cell envelope, likely due to the presence of the overlying OL and PL, its presence is consistent with our previous genomic analysis demonstrating an intact peptidoglycan biosynthesis pathway and is therefore considered to enclose the entire filament of cells (Sivabalasarma et al., 2025).

Archaellated cells displayed a polar localized archaellum utilising this remodelled polar envelope as an assembly platform. An additional density is localized between the inner layer and the cytoplasmic membrane, where the archaellum enters the cell (Figure 3B, white arrow). While the function of this feature cannot be definitively established from a single tomographic reconstruction, its localisation at the archaellum insertion site suggests a potential structural role in archaellum anchoring. However, examination of additional tomographic data would be necessary to confirm whether this represents a distinct anchoring subcomponent or an integral part of the archaellum itself.

In conclusion, we observed a multilayered cell envelope architecture with an outermost layer encapsulating all cells within a filament, while individual cells were enclosed by a cytoplasmic membrane and an inner layer. CryoET revealed that cell poles exhibited a distinct remodelled envelope lacking the outer and proteinaceous layer but retaining peptidoglycan. This specialised polar architecture most likely provides the platform for archaellum anchoring in archaellated cells. Together, these findings demonstrate that localized polar cell envelope remodelling is a key structural adaptation possibly enabling archaellum integration and stable anchoring within the multi-layered cell envelope of *L. aerophila*.

### 2.3 Additional cell surface appendages in *L. aerophila*

**Figure 4:**
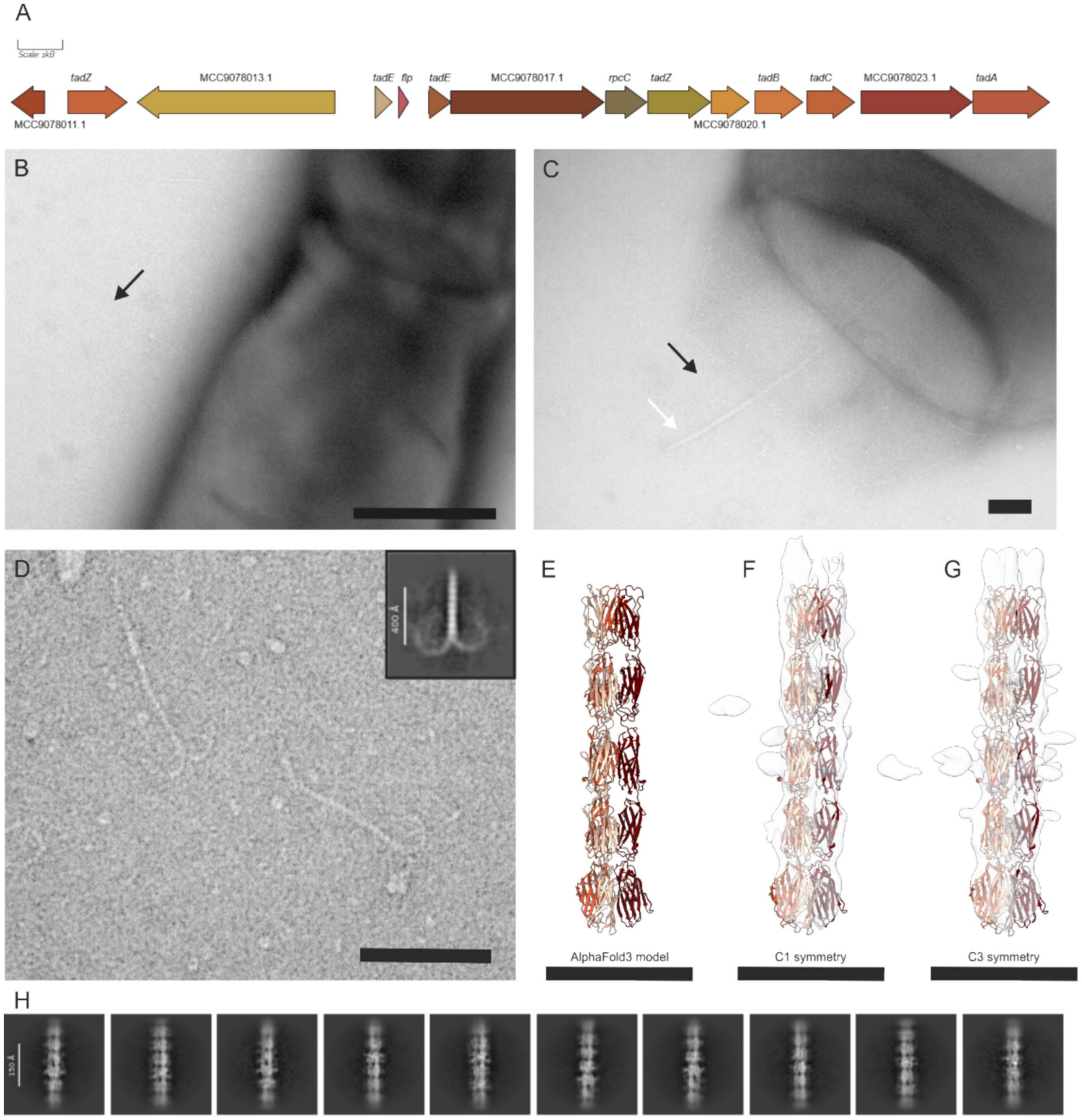
Cell surface appendages. **(A)** Genetic organization of the *tad* gene cluster. **(B)** Negative-stained TEM images showing a Tad pilus (black arrow) on the lateral side of *L. aerophila*. **(C)** Negative-stained TEM image of cell poles of *L. aerophila*. Grappling hook-like structures (black arrow) and an archaellum (white arrow) are visible. **(D)** Close-up of isolated grappling hook-like filaments imaged by TEM. An example 2D class average of negatively stained hooks is shown in the upper left corner. **(E)** Alphafold3 prediction of amino acid 2,347–2,954 of WP_141610714.1 corresponding to five DUF11 domains. The three different monomers are represented in different colors. **(F)** CryoEM volume from helical refinement job (white in surface representation), with no imposed symmetry (C1) and fitted AlphaFold3-model. **(G)** CryoEM volume from helical refinement job (white in surface representation), with imposed symmetry (C3) and fitted AlphaFold3-model. **(H)** 2D class averages of isolated hook filaments imaged by cryoEM. Scale Bars: 500 μm (B); 100 nm (C, D); 100 Å in panels **(E–G)**.

In addition to the archaellum, negative stain transmission electron microscopy of *L. aerophila* cells revealed additional cell surface-associated appendages. Thin, pilus-like structures were observed distributed along the lateral side of the cells (Figure 4B). Genomic analysis revealed the presence of a Tad-pilus operon including *tadA*, *tadB*, two copies of *tadZ*, *tadE*, *tadC* and one copy of *flp* (Figure 4A, Supplementary Table 1). No additional pilus biogenesis systems were found, suggesting that these cell appendages are tad pili (Sivabalasarma et al., 2025).

In addition to the tad pili, thicker filamentous structures were observed exclusively at the cell poles. These filamentous structures were approximately 180 nm in length and contained a grappling hook structure at their end (Figure 4C, black arrow). These resembled the grappling hook appendages recently described in *Aureispira* sp., which are formed by GhpA, a 5,898-residue monomer that spans the length of the filament and assembles as a heptamer (Lien et al., 2024). BLAST analysis of the *L. aerophila* genome identified a homologous sequence, WP_141610714.1, which we will refer to as grappling hook-like protein A (GhlA). GhlA is a 6,596 amino acid protein (672 kDa) containing 49 DUF11 domains. Structural predictions using AlphaFold3 revealed a long filamentous structure composed of the 49 DUF11 domains, arranged consecutively like beads on a string (Figure 4E). Notably, only 33 DUF11 domains are annotated on UniProt (ID: A0A540VE73_9CHLR). Each DUF11 domain consists of 10 antiparallel *β*-strands that assemble in two sheets, facing each other (Figure 4E). The structure closely resembles an immunoglobulin (Ig)-like fold, comprising 7–9 antiparallel β-strands arranged into two opposing sheets. The DUF11 domain-containing protein harbors a 19-residue hydrophobic stretch at its N-terminus, which is predicted to be a transmembrane domain. Notably, it lacks an N-terminal signal peptide, suggesting that its secretion occurs via an alternative, signal-peptide-independent pathway.

To verify the identity of the hook-forming protein, CsCl gradient fractions containing isolated hooks were analyzed by mass spectrometry. A total of 1,272 proteins were identified (Supplementary Table 2). GhlA (WP_141610714.1) was identified with 26 unique peptides spanning amino acids 257–6,566 of its predicted sequence (Supplementary Table 3) and ranked 38th in overall intensity (Supplementary Table 2). When proteins were ranked by relative weight fraction based on intensity-based absolute quantification (iBAQ), GhlA ranked 21st (Supplementary Figure 3). The abundance and peptide coverage of GhlA in the hook-containing fractions confirmed its identity as the hook-forming protein.

### 2.4 Structural analysis of the grappling hook filament

For structural analysis, hook filaments were vitrified and imaged by cryoEM (Figures 4C,D). Particle picking and classification yielded in 1.858.183 particles. Due to the high flexibility of the filamentous hook tip, we focused on the hook core and neglected the grappling hook region (Figure 4). Particle picking and classification yielded in 10 high-quality 2D classes (104,631 particles), which contained sharp, well-distinguishable features. The best 2D class averages achieved resolutions between 7 Å and 9 Å (Figure 4H). The DUF11 domains were distinctly resolved and, consistent with the structure prediction, appeared as beads on a string-like structure. Individual domains were tilted relatively to one another, potentially reflecting inter-domain flexibility. The DUF domains exhibited an O-shaped appearance, possibly arising from a gap between the opposing β- sheets within the IG-like fold. Furthermore, densities protruding from the filament were observed that could not be assigned to the previously AlphaFold3-predicted structure (Figures 4F,G). These are too large to represent glycan modifications and may correspond to proteinaceous features.

Ab initio 3D reconstruction and helical refinement produced a 6.75 Å volume (C1 symmetry), which indicated a three-stranded arrangement (Figure 4F). Imposing C3 symmetry improved resolution to 6.39 Å (Figure 4G). An AlphaFold3 model of three copies of residues 2,347–2,945 (five DUF-domains) fit well in both C1 and C3 volumes, supporting probable C3 symmetry of the hook filament stem (Figures 4F,G).

## 3 Discussion

*Litorilinea aerophila* grows in multicellular organized filaments (Figures 1, 2), consistent with the initial description (Kale et al., 2013). During growth in liquid culture, filaments transitioned from long, irregular segmented structures into shorter filaments (Figures 1B,C). Diversity in filament length has also been reported in cyanobacteria, where shorter motile filaments (hormogonia) differentiate in response to external signals to engage in symbiosis (Risser, 2023). In liquid cultures, longer *L. aerophila* filaments formed dense cell aggregates resembling early-stage biofilm structures (Figure 1). As growth continued, these filaments shortened and, as reported previously, became motile (Sivabalasarma et al., 2025). This points towards an analogous morphological transition in growth stages from nonmotile long filaments to shorter motile cells.

*L. aerophila* showed a multilayered cell envelope decorated with a variation of cell surface structures: Polar localized grabbling hook structures and archaella and lateral localized tad pili (Figure 4). Tad pili are known to mediate surface adhesion and biofilm formation, thus likely contribute to the observed aggregation behavior of *L. aerophila* in liquid culture (Figure 1B I.). The observed hook-like cell surface structures resemble the predatory grappling hooks described in *Aureispira* sp., which are implicated in ixotrophic behavior (Lien et al., 2024). Apart from bacteria, similar hook-like structures have also been found in archaea with quite a different functionality. These structures, called hami, are known to facilitate the formation of interconnected cell networks (Moissl et al., 2005; Probst et al., 2014). Even though visually hami tips in particular look very similar to *L. aerophila* hooks, no homologs of the hamus subunit protein (Moissl et al., 2005; Perras et al., 2015) could be found in *L. aerophila*.

**Figure 5:**
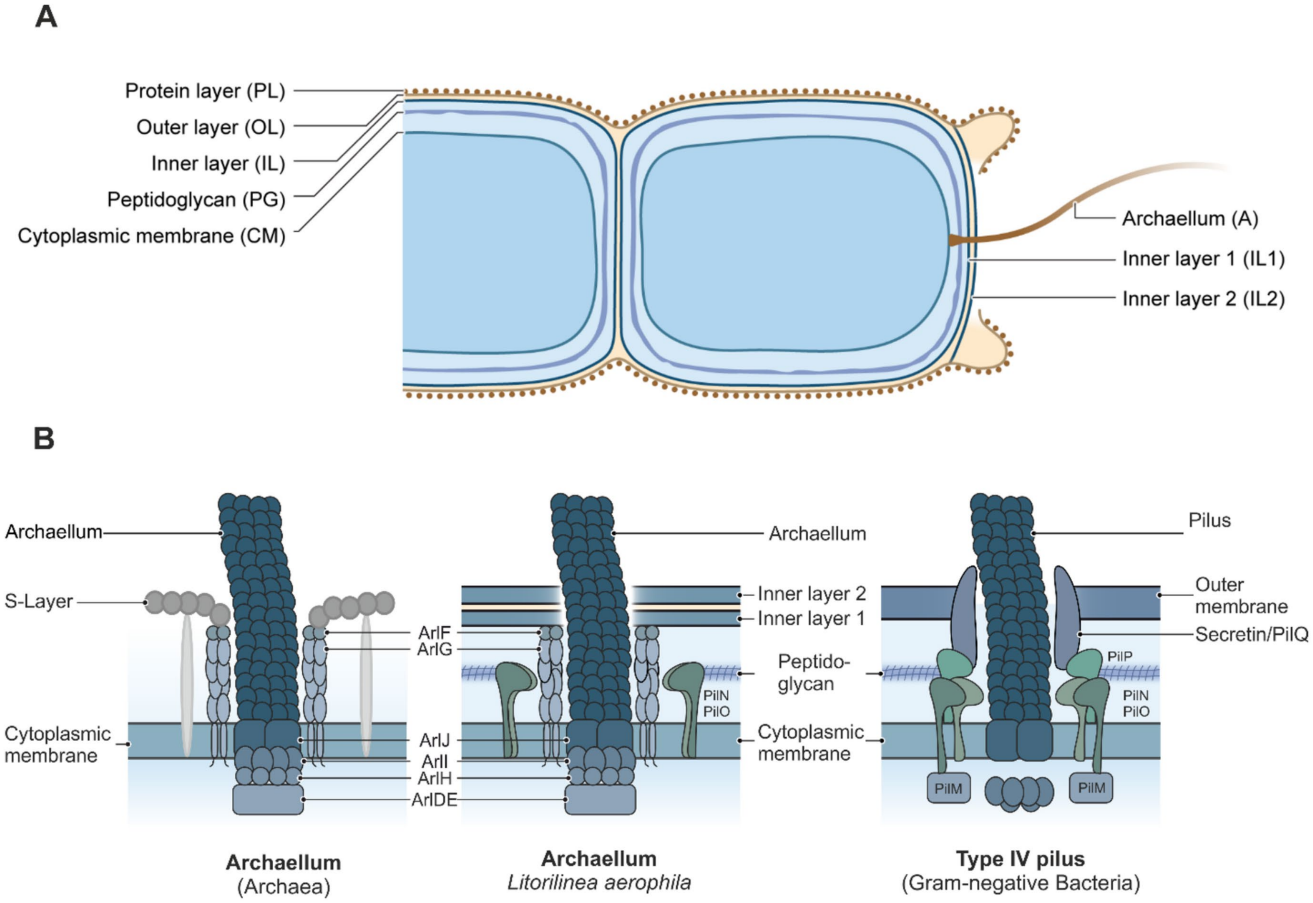
Model of the *L. aerophila* cell envelope and archaellum anchoring. **(A)** Multicellular filaments carry a multilayered cell envelope composed of a cytoplasmic membrane, followed by a peptidoglycan layer and an inner layer. These respective layers enclose individual cells within the filament. All cells are enclosed by a shared outer layer, decorated with a proteinaceous layer. The archaellum is polar localized. **(B)** A comparison of the archaellum in archaea and bacteria, and the type IV pilus in Gram-negative bacteria, and how it traverses the cell envelope.

Motile cells carried a polar localized archaellum (Figure 3A). The archaellum filament entered the cell pole, where an additional density was observed beneath the entry point between the inner layer and the cytoplasmic membrane (Figure 3B, white arrow). The identity of this feature is unclear and does not resemble the canonical archaellum core complex. Given the limited knowledge of archaellum expression in *L. aerophila* and the fact that this observation is based on a single tomogram, it should be interpreted with caution. Nevertheless, the localization of this density is consistent with a region where additional structural components could facilitate filament passage through the cell envelope. Notably, PilN- and PilO-homologs are encoded in the genetic neighborhood of the archaellum genes in *L. aerophila* (Figure 5B) (Sivabalasarma et al., 2025).

Type IV pili (T4P) in Gram-negative bacteria traverse the cell envelope via an alignment complex and an outer membrane secretin (Hospenthal et al., 2017). The secretin cage connects through the lipoprotein PilP to the PilN-PilO alignment complex (Figure 5B) (Tammam et al., 2013; Chang et al., 2016; Weaver et al., 2020; Guo et al., 2024). PilO and PilN are membrane proteins that form higher-order oligomeric cage-like structures spanning the inner membrane and bind to the peptidoglycan (Ayers et al., 2009; Sampaleanu et al., 2009). The N-terminus of PilN interacts with the cytoplasmic protein PilM (Karuppiah and Derrick, 2011). Together, PilQ/P/O/N/M form a continuous scaffold that guides the pilus across the cell envelope (Figure 5B) (Tammam et al., 2013; Chang et al., 2016; Craig et al., 2019).

In *L. aerophila*, homologs of *pilN* and *pilO* are present, whereas genes encoding for *pilQ*, *pilP* and *pilM* are absent, leading to the question of the possible role of PilN and PilO. In *Neisseria meningitidis*, a bacterial two hybrid study showed interaction of PilO with PilC, the membrane platform protein of the T4P (Georgiadou et al., 2012). Furthermore, in *Myxococcus xanthus* correct localization of PilC depends on PilO (Friedrich et al., 2014). We therefore hypothesize that PilN and PilO in *L. aerophila* form an alternative alignment scaffold guiding the archaellum through the multi-layered cell envelope (Figure 5B).

Interestingly, the *L. aerophila* PilO homolog encodes for a LysM domain, a peptidoglycan binding motif that differs from canonical T4P PilO proteins, where peptidoglycan anchoring is mediated by the separate TsaP protein (Siewering et al., 2014). To explore whether the PilO-LysM fusion might be relevant to archaellum anchoring to the cell envelope in Chloroflexota, a comprehensive genomic analysis of 244 archaellated Chloroflexota members was done. Indeed, from 244 archaellated members, 235 encode for genes for peptidoglycan biosynthesis and from those, 34 members encode for *pilO* and *pilN* (Supplementary Table 4). However, only 3 Chloroflexota organisms encoded *pilO* with a LysM domain in the genetic neighborhood of the archaellum genes (Supplementary Table 5). While the importance of this observation remains unclear, it may suggest that PilO-LysM fusion could represent an adaptation in some archaellum encoding Chloroflexota. The presence of PilO-LysM in the genetic neighbourhood of *L. aerophila* could potentially enable direct peptidoglycan binding by PilO, though the functional implications remain speculative.

However, how other members of the Phylum Chloroflexota encoding for peptidoglycan biosynthesis but lacking PilO and PilN anchor archaella is unknown. The remarkable structural diversity of the multi-layered cell envelope architecture across Chloroflexota suggests that multiple mechanisms may exist for integrating archaella into these unusual cell envelope types.

Additionally, interaction of PilO with the archaellum machinery could potentially influence correct localization of the archaellum (Figure 5B). Such a scaffold could facilitate the passage of the stator complex and filament through the peptidoglycan and enhance structural stability (Figure 5B). In *Helicobacter pylori*, *pilO*, *pilN* and *pilM* homologs form a cage-like scaffold around the flagellar motor that modulates motility, suggesting structural compatibility and possible exaptation of type IV pilus components for structurally distinct flagella and archaella (Liu et al., 2024).

While lateral cells are enclosed by a continuous outer layer spanning the multicellular filament, the polar region lacks this outer layer and instead displays a distinct inner layer organization and a clearly defined peptidoglycan (Figure 3A,D 5A). While the identity of these layers is unclear, Chloroflexota lack canonical outer membrane markers such as the BAM complex and lipopolysaccharide biosynthesis genes (Sutcliffe, 2010). The complex, multilayered cell envelope of *L. aerophila* therefore supports broader cell envelope diversity within the phylum (Figure 5A). Similar multilayered cell envelope architectures were recently described in other members of the phylum Chloroflexota, including *Ca.* Viridilinea mediisalina and *C. aggregans*, reflecting convergent evolutionary adaptations rather than homologous diderm envelopes (Gaisin et al., 2020).

Our model (Figure 5B) points towards an exaptation of the type IV pilus-specific PilN/PilO as an adaptation to the present multilayered cell envelope and peptidoglycan layer in *L. aerophila*. However, the mechanism by which the filament crosses the additional inner layers remains unresolved. Notably, even in archaea, the process by which the archaellum filament exits the proteinaceous surface layer is not understood.

In summary, our data show that the horizontally acquired archaellum machinery in *L. aerophila* is functionally integrated into a specialized polar region of a multilayered bacterial cell envelope. We propose that recruitment of the PilO/PilN alignment complex provides a scaffold that bridges the peptidoglycan, enabling the stable anchoring of the archaellum (Figure 5). This architecture reveals an unexpected level of structural flexibility in the bacterial cell envelope. Thus, our findings highlight how cross-domain gene transfer of a previously thought archaea-specific motility machinery is coupled with bacterial cell envelope remodeling, enabling its stable function in a bacterial context.

## 4 Methods

### 4.1 Bacterial strains

*Litorilinea aerophila* PRI 4131 (DSM 25763) was obtained from the DSMZ (German Collection of Microorganisms and Cell Cultures GmBH). *Litorilinea aerophila* PRI 4131 was previously isolated from an intertidal hot spring in Isafjardardjup, NW Iceland (Kale et al., 2013).

### 4.2 Growth evaluation

*Litorilinea aerophila* was grown in 5 mL Marine Broth (MB) (Difco 2,216) in plastic tubes. Cells were incubated at 55 °C with shaking at 90 rpm. For determination of the cell dry weight (mg ml^-1^) one 5 mL culture was inoculated for each measuring point to a theoretical OD 0.02. Cell were grown at 55 °C, 90 rpm shaking. Four milliliter of these cultures were pelleted, and the cells were washed once with PBS. The cell pellet was transferred to calibrated reaction tubes and dried in a centrifugal vacuum concentrator (SpeedVac™, ThermoFisher) at 45 °C for 1 h before measuring the weight of each pellet. The experiment was done in triplicate.

### 4.3 Light and fluorescence microscopy

0.5 μL Syto13 (ThermoFisher) and 1 μL Nile red (5 mg/mL in DMSO) (ThermoFisher) were added to 500 μL of each culture from the dry weight determination. Cells were incubated for 10 min in the dark at room temperature. Cells were washed once with PBS, followed by resuspension in 500 μL PBS. For imaging 5 μL of cells of spotted on an agarose pad made of 1% agar in PBS. Cells were observed at x100 magnification in the DIC mode using an inverted microscope (Zeiss Axio Observer. Z1, controlled via Zeiss Blue v.3.3.89 software). Image analysis was performed using ImageJ.

### 4.4 Negative stain electron microscopy

Five milliliter Marine Broth medium was inoculated with *L. aerophila* and grown overnight at 55 °C, 90 rpm shaking. Samples were taken from day 1,2,3 and 5 μL of cells were applied on freshly glow-discharged carbon/Formvar-coated copper grids (300 mesh; Plano GmbH) and incubated for 30 s. The excess liquid was blotted away and the grid washed three times with double distilled water (ddH_2_O), followed by negative staining with 2% uranyl acetate. Images were taken with a Hitachi HT7800 operated at 100 kV, equipped with an EMSIS Xarosa 20-megapixel CMOS camera.

### 4.5 Thin-section preparation for electron microscopy

Five milliliter of cell culture were centrifuged for 5 min at 5.000 rpm. The cell pellet was resuspended in 50 μL of 2% (w/v) Ultra-low Gelling Temperature Agarose (SIGMA A-2576) and let solidify at 4 °C for 5 min. Cells embedded in the agarose were fixed in a 2.5% (v/v) glutaraldehyde solution in MTSB Buffer (50 mM PIPES, 5 mM EGTA, 5 mM MgSO_4_·7H_2_O, 50 mM KOH) for 5 h at room temperature and overnight at 4 °C with fresh fixative solution. Samples were washed with MTSB five times (10 min each) and post fixed in aqueous 1% (w/v) OsO_4_ on ice during 3 h. Samples were washed at room temperature with water five times, each for 10 min, and *in bloc* stained with 2% UrAc (w/v) in water for 2 h. After washing the samples twice with water for 5 min, they were dehydrated by incubation for 15 min in increasing EtOH/water graded series (30%. 50, 70, 80, 95%) and for 30 min twice in 100% EtOH and 100% Acetone. Samples were gradually embedded in resin by incubating them in Agar 100:acetone mixtures (1:3, 1:1, 3:1) 8 h each, and finally inpure resin (3 times exchange, 8 h each). Resin blocks containing the embedded cells were polymerized for 2 days at 60 °C and 70 nm sections were obtained with a Reichert-Jung Ultracut-E microtome. Sections were collected on copper grids, contrasted with 2% uranyl acetate and Reynolds lead citrate solution(Reynolds, 1963) and observed in a Hitachi 7,800 TEM coupled to a Xarosa CMOS camera (Emsis).

### 4.6 Purification of cell surface filaments

A preculture was grown by inoculating a single colony of *Litorilinea aerophila* into 5 mL MB and incubating overnight at 55 °C with gentle shaking (90 rpm). The following day, 1 mL of culture was transferred into 2 × 50 mL MB in 250 mL Erlenmeyer flasks and grown overnight under the same conditions. The OD₆₀₀ was measured and diluted to ~0.01–0.02 into 4–6 × 400 mL MB in 2 L flasks, followed by incubation at 55 °C for 5 days with 90 rpm shaking. Cells were harvested by centrifugation (3,000 × g, 30 min), resuspended in 50 mL isolation buffer (1x PBS + 2% NaCl), and subjected to hook shearing using a peristaltic pump: 1 h through a 0.9 mm × 40 mm needle, then 2 h through a 0.45 mm × 10 mm needle, both at 90 rpm. Sheared cells were removed by low-speed centrifugation (12,000 × g, 25 min, 4 °C), and the supernatant was ultracentrifuged (200,000 × g, 1 h 20 min, 4 °C). The resulting pellet was resuspended in 500 μL isolation buffer and layered onto a 4.5 mL CsCl gradient (0.5 g/mL in isolation buffer), followed by overnight centrifugation (250,000 × g, 16 h, 4 °C). Five milliter fractions were collected, diluted to 8 mL with isolation buffer, and pelleted again (250,000 × g, 1.5 h, 4 °C). Pellets were resuspended in 50 μL isolation buffer and analysed by negative-stain TEM to identify hook-rich fractions. Selected fractions were concentrated to 25 μL using 100 kDa Amicon filters (Sigma Aldrich).

### 4.7 Cryo-electron microscopy sample preparation and data acquisition

R2/2 copper grids (Quantifoil Micro Tools) were glow discharged and 3.5 μL of isolated hook filaments were applied. The grids were vitrified in liquid ethane and stored in liquid nitrogen. The dataset was collected with a Titan Krios electron microscope (Thermo Fisher), equipped with a Selectris energy filter (Gatan) and a Falcon 4i electron detector (Thermo Fisher). For data collection the pixel size was calibrated to 1.22 Å, corresponding to a magnification of 105,000 x. Using EPU 3.6 software (Thermo Fisher), 11,060 movies were collected as 40-fraction movies with an exposure time of 5.85 s, a total electron dose of 40 e- / Å 2 and a defocus range of −0.5 to −2.2 μm.

### 4.8 Processing of the hook filaments dataset in CryoSPARC

Image processing was performed in CryoSPARC v4.6 (Punjani et al., 2017). Motion correction and CTF estimation were done using the patch-based methods. An initial set of 30 manually picked micrographs was used to train a CrYOLO filament-picking model in filament mode (Wagner et al., 2019; Wagner et al., 2020), resulting in 1,858,183 automatically picked particles. Particles were extracted with a 300 px box size and Fourier-cropped to 100 px for four rounds of 2D classification (40 iterations, 100 classes, batch size 500). From this, 519,123 particles across 78 classes were selected and re-extracted at full resolution. An initial 3D model was generated ab initio from the sharpest 104,631 particles (10 classes) using C1 symmetry and windowing (0.75–0.9), with minibatch sizes of 300–1,000. This was refined using helical refinement first with C1 (6.75 Å), then C3 symmetry, supported by fitting an AlphaFold-predicted trimer (WP_141610714.1) (Jumper et al., 2021). The full 519,123-particle set was then refined using this C3 volume, improving the resolution to 5.05 Å. To explore heterogeneity, 3D classification (five classes, 7 Å filter, C3 symmetry, hard classification) was followed by helical refinement of each class (5.97–6.23 Å, ~86 k–128 k particles). The best class was further classified and refined, but yielded lower-resolution volumes (7.1–7.53 Å, ~17 k–26 k particles). A strand-specific mask was generated in ChimeraX (Goddard et al., 2018) and applied in a helical refinement (C1, 519,123 particles), yielding only minor resolution gains. Attempts to loosen the mask via threshold/dilation worsened the results.

### 4.9 Mass spectrometry sample preparation, data acquisition, and analysis

Proteins were denatured in 1% SDS and cysteine residues were reduced with 10 mM DTT for 15 min at 95 °C. For alkylation of free thiol groups 50 mM chloroacetamide was added for 30 min at 22 °C. The reaction was quenched with 50 mM DTT. Samples were purified and digested with trypsin in a 96-deep-well plate (1 mL, Agilent Technologies) with an SP3-bead-based protocol (Hughes et al., 2019) adapted to a STARlet automated liquid handling system (Hamilton, Bonaduz Switzerland) equipped with a 96-well magnet plate (Magnum Flex, Alpaqua). Proteins were bound to 100 μg Sera-Mag SpeedBead magnetic carboxylate-modified particles (Cytiva) with 80% ethanol for 20 min at room temperature and washed three times with 80% ethanol. Proteins were digested overnight at 37 °C in 50 μL of 100 mM ammonium bicarbonate containing 0.15 μg of trypsin (Promega V5111, Madison, USA). Digestion was stopped by adding 30 μL of 5% formic acid (FA). Peptides were desalted using self-packed SDB-RPS Stop and Go Extraction tips (StageTips) (Rappsilber et al., 2007) composed of three layers of 1.0 × 1.0 mm (AttractSPE® Disks Bio RPS, Affinisep). The StageTips were equilibrated sequentially with 100% methanol, 80% acetonitrile containing 0.1% FA, and twice with 0.1% FA. Acidified peptide solutions were loaded onto the StageTips and washed with 0.1% FA, 80% acetonitrile with 0.1% FA, and 80% methanol with 0.5% FA. Peptides were eluted using 60% acetonitrile containing 5% ammonia, dried in a vacuum centrifuge and reconstituted in 0.1% TFA.

LC–MS analysis was carried out on an UltiMate 3,000 RSLCnano system coupled to a Q Exactive mass spectrometer (both Thermo Fisher Scientific) as described (Bhuiyan et al., 2025)with minor changes. In brief, peptides were preconcentrated on a PepMap C18 trapping column and separated on a μPAC™ C18 pillar array column (200 cm bed length, Thermo Fisher Scientific) applying a binary buffer system consisting of A (0.1% formic acid) and B (86% acetonitrile, 0.1% formic acid) and a linear gradient from 5 to 50% B in 45 min and 50 to 99% B in 2 min at a flow rate of 1 μL/min. Cycles of data-independent acquisition (DIA) consisted of: one overview spectrum (RF lens of 50%, normalized AGC target of 3e6, maximum injection time of 60 ms, *m/z* range of 385 to 1.050, resolution of 70.000 at *m/z* 200, profile mode) followed by MS2 fragment spectra (RF lens of 50%, normalized AGC target of 1e6, maximum injection time of 60 ms) generated sequentially by higher-energy collision-induced dissociation (HCD) at a normalized energy of 26% in a precursor *m/z* range from 400 to 1.000 in 50 windows of 12.5 *m/z* isolation width overlapping by 0.25 *m/z* and recorded at a resolution of 35.000 in profile mode.

Mass spectrometric raw data were converted to mzML format using the ProteoWizard software, version 3.0.21229 (Chambers et al., 2012). For protein and peptide identification a library free search was performed using FragPipe, version 22.0 (Yu et al., 2023) with quantification by Dia-NN, version 1.82 (Demichev et al., 2020) against the organism specific sequence database for *Litorilinea aerophila* from Uniprot database (release 2025_02, 4,877 entries). Trypsin was set as protease with 2 allowed missed cleavages. Carbamidomethylation of cysteine was selected as fixed modification, N-terminal excision of methionine, oxidation of methionine and N-terminal acetylation were selected as variable modifications with a maximum number of 3. Further parameters were a 1% protein false discovery rate cutoff, unrelated runs and robust LC quantification. All protein groups with non-zero intensities listed in the protein and protein group output matrices of DIA-NN were selected for further analysis. Intensity-based absolute quantification (iBAQ) values were calculated by dividing the sum of peptide intensities by the theoretical number of peptides predicted for proteolytic digestion by trypsin, considering peptides in the range of 7 to 35 amino acids that typically detected during LC–MS analysis (Schwanhäusser et al., 2011). Relative weight fractions were derived by multiplying iBAQ values for each protein with its predicted molecular weight and normalizing to the sum across all proteins.

#### 4.9.1 Cryo-electron tomography

*L. aerophila* culture was grown to OD_600_ ~ 0.9 in liquid marine broth medium at 55 °C and 90 rpm. A total of 3.5 μL cells were applied on freshly glow-discharged EM copper grids (R2/2, Quantifoil) and plunge frozen in liquid ethane using the Leica GP2. Frozen grids were stored in liquid nitrogen. CryoET data was collected using a Titan Krios (Thermo Fisher Scientific) cryo electron microscope operating at 300 kV equipped with a Falcon4i and Selectris energy filter (Gatan) at a calibrated pixel size of 4.71 Å corresponding to 26,000x magnification. Using Tomography 5 (Thermo Fisher Scientific) 12 tilt series were collected with a tilt range of +60° to −60° in 3° increments, a total electron dose of 140 e^−^/ Å^2^ and a defocus range from −5 to −8 μm. Tilt series were driftcorrected using alignframes in IMOD (Kremer et al., 1996). Corrected tilt series of the non-motile cells were used to reconstruct 4× binned cryo-tomograms with IMOD using weighted back projection. Tomograms were CTF-deconvolved and filtered using isonet (Liu et al., 2022). Corrected tilt series of the motile cells were used to reconstruct 4× binned cryo-tomograms with AreTomo3 using weighted back projection (Peck et al., 2025). Tomograms were CTF-deconvolved and filtered using isonet2 (Liu et al., 2025).

#### 4.9.2 Identification of peptidoglycan biosynthesis genes

Cell division and PG biosynthesis related cluster was identified using Macsyfinder V2 (Abby et al., 2024) with a custom model specific to cell division and peptidoglycan related proteins. A total of 244 Chloroflexota genomes encoding for an archaellum machinery from Sivabalasarma et al., 2025 were screened using a custom MacSyFinder model. Custom model was generated with a curated set of HMM profiles derived from KEGG (Kanehisa and Goto, 2000; Kanehisa, 2019; Kanehisa et al., 2025) orthology related to peptidoglycan biosynthesis (map00550). Macsyfinder was run with the custom model in ordered replicon mode, with all genes set as accessory. PilO and PilN homologs were identified through NCBI BLASTP webserver search using *Litorilinea aerophila* PilO and PilN as a query sequence.

## Data Availability

The mass spectrometry proteomics data have been deposited to the ProteomeXchange Consortium via the PRIDE partner repository (Perez-Riverol et al., 2025) with the dataset identifier PXD066799 (Reviewer access details: Project accession: PXD066799; Token: mz0A1Tb4XsDJ). The cryoEM map have been deposited in the EMDB under accession numbers EMD-54772 (C1 symmetry) and EMD-54773 (C3 symmetry). The cryo-electron tomograms have been deposited in the EMDB under accession number EMD-54781 (cell pole), EMD-57253 (archaellated cell pole) and EMD-5780 (cell).

## References

[ref1] Abby S. S. Denise R. Rocha E. P. C. (2024). “Identification of protein secretion Systems in Bacterial Genomes Using MacSyFinder version 2,” in Bacterial Secretion Systems: Methods and Protocols, eds. Journet L. Cascales E. (New York, NY: Springer US), 1–25. 10.1007/978-1-0716-3445-5_1 37930518

[ref2] Albers S.-V. Jarrell K. F. (2018). The Archaellum: an update on the unique archaeal motility structure. Trends Microbiol. 26, 351–362. doi: 10.1016/j.tim.2018.01.004, 29452953

[ref3] Ayers M. Sampaleanu L. M. Tammam S. Koo J. Harvey H. Howell P. L. . (2009). PilM/N/O/P proteins form an inner membrane complex that affects the stability of the *Pseudomonas aeruginosa* type IV pilus secretin. J. Mol. Biol. 394, 128–142. doi: 10.1016/j.jmb.2009.09.034, 19857645

[ref4] Banerjee A. Tsai C.-L. L. Chaudhury P. Tripp P. Arvai A. S. Ishida J. P. . (2015). FlaF is a b-sandwich protein that anchors the archaellum in the archaeal cell envelope by binding the S-layer protein. Structure 23, 863–872. doi: 10.1016/j.str.2015.03.001, 25865246 PMC4425475

[ref5] Beeby M. Ferreira J. L. Tripp P. Albers S.-V. Mitchell D. R. (2020). Propulsive nanomachines: the convergent evolution of archaella, flagella and cilia. FEMS Microbiol. Rev. 44, 253–304. doi: 10.1093/femsre/fuaa006, 32149348

[ref6] Benyahia B. B. Taib N. Beloin C. Gribaldo S. (2025). Terrabacteria: redefining bacterial envelope diversity, biogenesis and evolution. Nat. Rev. Microbiol. 23, 41–56. doi: 10.1038/s41579-024-01088-0, 39198708

[ref7] Bhuiyan T. Arecco N. Mendoza Sanchez P. K. Kim J. Schwan C. Weyrauch S. . (2025). TAF2 condensation in nuclear speckles links basal transcription factor TFIID to RNA splicing factors. Cell Rep. 44:115616. doi: 10.1016/j.celrep.2025.115616, 40287942

[ref8] Chambers M. C. Maclean B. Burke R. Amodei D. Ruderman D. L. Neumann S. . (2012). A cross-platform toolkit for mass spectrometry and proteomics. Nat. Biotechnol. 30, 918–920. doi: 10.1038/nbt.2377, 23051804 PMC3471674

[ref9] Chang Y. W. Rettberg L. A. Treuner-Lange A. Iwasa J. Søgaard-Andersen L. Jensen G. J. (2016). Architecture of the type IVa pilus machine. Science 351:aad2001. doi: 10.1126/science.aad2001, 26965631 PMC5929464

[ref10] Craig L. Forest K. T. Maier B. (2019). Type IV pili: dynamics, biophysics and functional consequences. Nat. Rev. Microbiol. 17, 429–440. doi: 10.1038/s41579-019-0195-4, 30988511

[ref11] Demichev V. Messner C. B. Vernardis S. I. Lilley K. S. Ralser M. (2020). DIA-NN: neural networks and interference correction enable deep proteome coverage in high throughput. Nat. Methods 17, 41–44. doi: 10.1038/s41592-019-0638-x, 31768060 PMC6949130

[ref12] Denise R. Abby S. S. Rocha E. P. C. (2019). Diversification of the type IV filament superfamily into machines for adhesion, protein secretion, DNA uptake, and motility. PLoS Biol. 17:e3000390. doi: 10.1371/journal.pbio.3000390, 31323028 PMC6668835

[ref13] Friedrich C. Bulyha I. Søgaard-Andersen L. (2014). Outside-in assembly pathway of the type IV pilus system in *Myxococcus xanthus*. J. Bacteriol. 196, 378–390. doi: 10.1128/JB.01094-13, 24187092 PMC3911261

[ref14] Gaisin V. A. Kooger R. Grouzdev D. S. Gorlenko V. M. Pilhofer M. (2020). Cryo-electron tomography reveals the complex ultrastructural organization of multicellular filamentous Chloroflexota (Chloroflexi) bacteria. Front. Microbiol. 11:1373. doi: 10.3389/fmicb.2020.01373/full, 32670237 PMC7332563

[ref15] Georgiadou M. Castagnini M. Karimova G. Ladant D. Pelicic V. (2012). Large-scale study of the interactions between proteins involved in type IV pilus biology in *Neisseria meningitidis*: characterization of a subcomplex involved in pilus assembly. Mol. Microbiol. 84, 857–873. doi: 10.1111/j.1365-2958.2012.08062.x, 22486968

[ref16] Goddard T. D. Huang C. C. Meng E. C. Pettersen E. F. Couch G. S. Morris J. H. . (2018). UCSF ChimeraX: meeting modern challenges in visualization and analysis. Protein Sci. 27, 14–25. doi: 10.1002/pro.3235, 28710774 PMC5734306

[ref17] Guo S. Chang Y. Brun Y. V. Howell P. L. Burrows L. L. Liu J. (2024). PilY1 regulates the dynamic architecture of the type IV pilus machine in *Pseudomonas aeruginosa*. Nat. Commun. 15:9382. doi: 10.1038/s41467-024-53638-y, 39477930 PMC11525922

[ref18] Hanada S. (2014). “The phylum Chloroflexi, the family Chloroflexaceae, and the related phototrophic families Oscillochloridaceae and Roseiflexaceae,” in The Prokaryotes: Other Major Lineages of Bacteria and the Archaea, eds. Rosenberg E. DeLong E. F. Lory S. Stackebrandt E. Thompson F. (Berlin, Heidelberg: Springer), 515–532.

[ref19] Hospenthal M. K. Costa T. R. D. Waksman G. (2017). A comprehensive guide to pilus biogenesis in gram-negative bacteria. Nat. Rev. Microbiol. 15, 365–379. doi: 10.1038/nrmicro.2017.40, 28496159

[ref20] Hug L. A. Castelle C. J. Wrighton K. C. Thomas B. C. Sharon I. Frischkorn K. R. . (2013). Community genomic analyses constrain the distribution of metabolic traits across the Chloroflexi phylum and indicate roles in sediment carbon cycling. Microbiome 1:22. doi: 10.1186/2049-2618-1-22, 24450983 PMC3971608

[ref21] Hughes C. S. Moggridge S. Müller T. Sorensen P. H. Morin G. B. Krijgsveld J. (2019). Single-pot, solid-phase-enhanced sample preparation for proteomics experiments. Nat. Protoc. 14, 68–85. doi: 10.1038/s41596-018-0082-x, 30464214

[ref22] Jumper J. Evans R. Pritzel A. Green T. Figurnov M. Ronneberger O. . (2021). Highly accurate protein structure prediction with AlphaFold. Nature 596, 583–589. doi: 10.1038/s41586-021-03819-2, 34265844 PMC8371605

[ref23] Kale V. Björnsdóttir S. H. Friðjónsson Ó. H. Pétursdóttir S. K. Ómarsdóttir S. Hreggviðsson G. Ó. (2013). *Litorilinea aerophila* gen. Nov., sp. nov., an aerobic member of the class Caldilineae, phylum Chloroflexi, isolated from an intertidal hot spring. Int. J. Syst. Evol. Microbiol. 63, 1149–1154. doi: 10.1099/ijs.0.044115-0, 22771681

[ref24] Kanehisa M. (2019). Toward understanding the origin and evolution of cellular organisms. Protein Sci. 28, 1947–1951. doi: 10.1002/pro.3715, 31441146 PMC6798127

[ref25] Kanehisa M. Furumichi M. Sato Y. Matsuura Y. Ishiguro-Watanabe M. (2025). KEGG: biological systems database as a model of the real world. Nucleic Acids Res. 53, D672–D677. doi: 10.1093/nar/gkae909, 39417505 PMC11701520

[ref26] Kanehisa M. Goto S. (2000). KEGG: Kyoto encyclopedia of genes and genomes. Nucleic Acids Res. 28, 27–30. doi: 10.1093/nar/28.1.27, 10592173 PMC102409

[ref27] Karuppiah V. Derrick J. P. (2011). Structure of the PilM-PilN inner membrane type IV pilus biogenesis complex from *Thermus thermophilus*. J. Biol. Chem. 286, 24434–24442. doi: 10.1074/jbc.M111.243535, 21596754 PMC3129222

[ref28] Kremer J. R. Mastronarde D. N. McIntosh J. R. (1996). Computer visualization of three-dimensional image data using IMOD. J. Struct. Biol. 116, 71–76. doi: 10.1006/jsbi.1996.0013, 8742726

[ref29] Lassak K. Neiner T. Ghosh A. Klingl A. Wirth R. Albers S.-V. (2012). Molecular analysis of the crenarchaeal flagellum. Mol. Microbiol. 83, 110–124. doi: 10.1111/j.1365-2958.2011.07916.x, 22081969

[ref30] Lien Y.-W. Amendola D. Lee K. S. Bartlau N. Xu J. Furusawa G. . (2024). Mechanism of bacterial predation via ixotrophy. Science 386:eadp0614. doi: 10.1126/science.adp0614, 39418385

[ref31] Lim Y. Seo J.-H. Giovannoni S. J. Kang I. Cho J.-C. (2023). Cultivation of marine bacteria of the SAR202 clade. Nat. Commun. 14:5098. doi: 10.1038/s41467-023-40726-8, 37607927 PMC10444878

[ref32] Liu Y.-T. Fan H. Jih J. Tran L. Zhang X. Zhou Z.H. (2025) IsoNet2 determines cellular structures at submolecular resolution without averaging. 2025.12.09.693325 doi: 10.64898/2025.12.09.693325v1.

[ref33] Liu X. Tachiyama S. Zhou X. Mathias R. A. Bonny S. Q. Khan M. F. . (2024). Bacterial flagella hijack type IV pili proteins to control motility. Proc. Natl. Acad. Sci. 121:e2317452121. doi: 10.1073/pnas.2317452121, 38236729 PMC10823254

[ref34] Liu Y.-T. Zhang H. Wang H. Tao C.-L. Bi G.-Q. Zhou Z. H. (2022). Isotropic reconstruction for electron tomography with deep learning. Nat. Commun. 13:6482. doi: 10.1038/s41467-022-33957-8, 36309499 PMC9617606

[ref35] Moissl C. Rachel R. Briegel A. Engelhardt H. Huber R. (2005). The unique structure of archaeal “hami”, highly complex cell appendages with nano-grappling hooks. Mol. Microbiol. 56, 361–370. doi: 10.1111/j.1365-2958.2005.04294.x, 15813730

[ref36] Peck A. Yu Y. Paraan M. Kimanius D. Ermel U.H. Hutchings J. . (2025) AreTomoLive: automated reconstruction of comprehensively-corrected and denoised cryo-electron tomograms in real-time and at high throughput. 2025.03.11.642690 doi: 10.1101/2025.03.11.642690v1. PMC13259924 42185541

[ref37] Perez-Riverol Y. Bandla C. Kundu D. J. Kamatchinathan S. Bai J. Hewapathirana S. . (2025). The PRIDE database at 20 years: 2025 update. Nucleic Acids Res. 53, D543–D553. doi: 10.1093/nar/gkae1011, 39494541 PMC11701690

[ref38] Perras A. K. Daum B. Ziegler C. Takahashi L. K. Ahmed M. Wanner G. . (2015). S-layers at second glance? Altiarchaeal grappling hooks (hami) resemble archaeal S-layer proteins in structure and sequence. Front. Microbiol. 6:543. doi: 10.3389/fmicb.2015.00543, 26106369 PMC4460559

[ref39] Probst A. J. Weinmaier T. Raymann K. Perras A. Emerson J. B. Rattei T. . (2014). Biology of a widespread uncultivated archaeon that contributes to carbon fixation in the subsurface. Nat. Commun. 5:5497. doi: 10.1038/ncomms6497 25425419

[ref40] Punjani A. Rubinstein J. L. Fleet D. J. Brubaker M. A. (2017). CryoSPARC: algorithms for rapid unsupervised cryo-EM structure determination. Nat. Methods 14, 290–296. doi: 10.1038/nmeth.4169, 28165473

[ref41] Rappsilber J. Mann M. Ishihama Y. (2007). Protocol for micro-purification, enrichment, pre-fractionation and storage of peptides for proteomics using StageTips. Nat. Protoc. 2, 1896–1906. doi: 10.1038/nprot.2007.261, 17703201

[ref42] Reynolds E. S. (1963). The use of lead citrate at high pH as an electron-opaque stain in electron microscopy. J. Cell Biol. 17, 208–212. doi: 10.1083/jcb.17.1.208, 13986422 PMC2106263

[ref43] Risser D. D. (2023). Hormogonium development and motility in filamentous cyanobacteria. Appl. Environ. Microbiol. 89, e00392–e00323. doi: 10.1128/aem.00392-23, 37199640 PMC10304961

[ref44] Sampaleanu L. M. Bonanno J. B. Ayers M. Koo J. Tammam S. Burley S. K. . (2009). Periplasmic domains of *Pseudomonas aeruginosa* PilN and PilO form a stable heterodimeric complex. J. Mol. Biol. 394, 143–159. doi: 10.1016/j.jmb.2009.09.037, 19857646

[ref45] Schwanhäusser B. Busse D. Li N. Dittmar G. Schuchhardt J. Wolf J. . (2011). Global quantification of mammalian gene expression control. Nature 473, 337–342. doi: 10.1038/nature10098, 21593866

[ref46] Siewering K. Jain S. Friedrich C. Webber-Birungi M. T. Semchonok D. A. Binzen I. . (2014). Peptidoglycan-binding protein TsaP functions in surface assembly of type IV pili. Proc. Natl. Acad. Sci. USA. 111, E953–E961. doi: 10.1073/pnas.1322889111 24556993 PMC3956165

[ref47] Sivabalasarma S. Taib N. Mollat C. L. Joest M. Steimle S. Gribaldo S. . (2025). Structure of a functional archaellum in Bacteria of the Chloroflexota phylum. Nat. Microbiol. 10, 2412–2424. doi: 10.1038/s41564-025-02110-8, 40962902 PMC12488501

[ref48] Sutcliffe I. C. (2010). A phylum level perspective on bacterial cell envelope architecture. Trends Microbiol. 18, 464–470. doi: 10.1016/j.tim.2010.06.005, 20637628

[ref49] Sutcliffe I. C. (2011). Cell envelope architecture in the Chloroflexi: a shifting frontline in a phylogenetic turf war. Environ. Microbiol. 13, 279–282. doi: 10.1111/j.1462-2920.2010.02339.x, 20860732

[ref50] Tammam S. Sampaleanu L. M. Koo J. Manoharan K. Daubaras M. Burrows L. L. . (2013). PilMNOPQ from the *Pseudomonas aeruginosa* type IV pilus system form a Transenvelope protein interaction network that interacts with PilA. J. Bacteriol. 195, 2126–2135. doi: 10.1128/JB.00032-13, 23457250 PMC3650547

[ref51] Tsai C.-L. Tripp P. Sivabalasarma S. Zhang C. Rodriguez-Franco M. Wipfler R. L. . (2020). The structure of the periplasmic FlaG–FlaF complex and its essential role for archaellar swimming motility. Nat. Microbiol. 5, 216–225. doi: 10.1038/s41564-019-0622-3, 31844299 PMC6952060

[ref52] Umrekar T. R. Winterborn Y. B. Sivabalasarma S. Brantl J. Albers S. V. Beeby M. (2021). Evolution of Archaellum rotation involved invention of a Stator complex by duplicating and modifying a Core component. Front. Microbiol. 12, 773386–773386. doi: 10.3389/fmicb.2021.773386, 34912317 PMC8667602

[ref53] van der Meer M. T. Schouten S. Hanada S. Hopmans E. C. Damsté J. S. Ward D. M. (2002). Alkane-1,2-diol-based glycosides and fatty glycosides and wax esters in *Roseiflexus castenholzii* and hot spring microbial mats. Arch. Microbiol. 178, 229–237. doi: 10.1007/s00203-002-0449-8, 12189424

[ref54] Wagner T. Lusnig L. Pospich S. Stabrin M. Schönfeld F. Raunser S. (2020). Two particle-picking procedures for filamentous proteins: SPHIRE-crYOLO filament mode and SPHIRE-STRIPER. Acta Crystallogr D Struct Biol 76, 613–620. doi: 10.1107/S2059798320007342, 32627734 PMC7336381

[ref55] Wagner T. Merino F. Stabrin M. Moriya T. Antoni C. Apelbaum A. . (2019). SPHIRE-crYOLO is a fast and accurate fully automated particle picker for cryo-EM. Commun. Biol. 2, 1–13. doi: 10.1038/s42003-019-0437-z, 31240256 PMC6584505

[ref56] Weaver S. J. Ortega D. R. Sazinsky M. H. Dalia T. N. Dalia A. B. Jensen G. J. (2020). CryoEM structure of the type IVa pilus secretin required for natural competence in *Vibrio cholerae*. Nat. Commun. 11:5080. doi: 10.1038/s41467-020-18866-y, 33033258 PMC7545093

[ref57] Yu F. Teo G. C. Kong A. T. Fröhlich K. Li G. X. Demichev V. . (2023). Analysis of DIA proteomics data using MSFragger-DIA and FragPipe computational platform. Nat. Commun. 14:4154. doi: 10.1038/s41467-023-39869-5, 37438352 PMC10338508

