# Supplementary material for: A multilayered cell envelope of a member of the Chloroflexota offers an anchoring platform for the archaellum
Source: Front Microbiol. 2026 Jun 17;17:1850455. doi: 10.3389/fmicb.2026.1850455 (PMC13319060; doi:10.3389/fmicb.2026.1850455)
Supplement: Supplementary file 1 [file Data_Sheet_1.PDF]

Supplementary material to

A multilayered cell envelope of a member of the Chloroflexota offers an anchoring platform for the Archaellum

Marie Joest[1,2], Clara L. Mollat[1], Laura Mutschler[1], Marta Rodriguez-Franco[3], Shamphavi Sivabalasarma[1,2], Friedel Drepper[4], Pitter F. Huesgen[4,5], Thomas Ott[3,5], Sonja-Verena Albers[1,5]

[1] Molecular Biology of Archaea, Faculty of Biology, University of Freiburg, 79104 Freiburg, Germany

[2] Spemann Graduate School of Biology and Medicine, University of Freiburg, 79104 Freiburg, Germany

[3] Cell Biology, Institute of Biology, Faculty of Biology, University of Freiburg, 79104 Freiburg, Germany

[4] Faculty of Biology, University of Freiburg, 79104 Freiburg, Germany

[5] CIBSS – Centre for Integrative Biological Signaling Studies

Supplementary Movie 1. Time-lapse microscopy of *L. aerophila* over 57 hours showing cell growth and breakage leading to shortening of multicellular filaments.

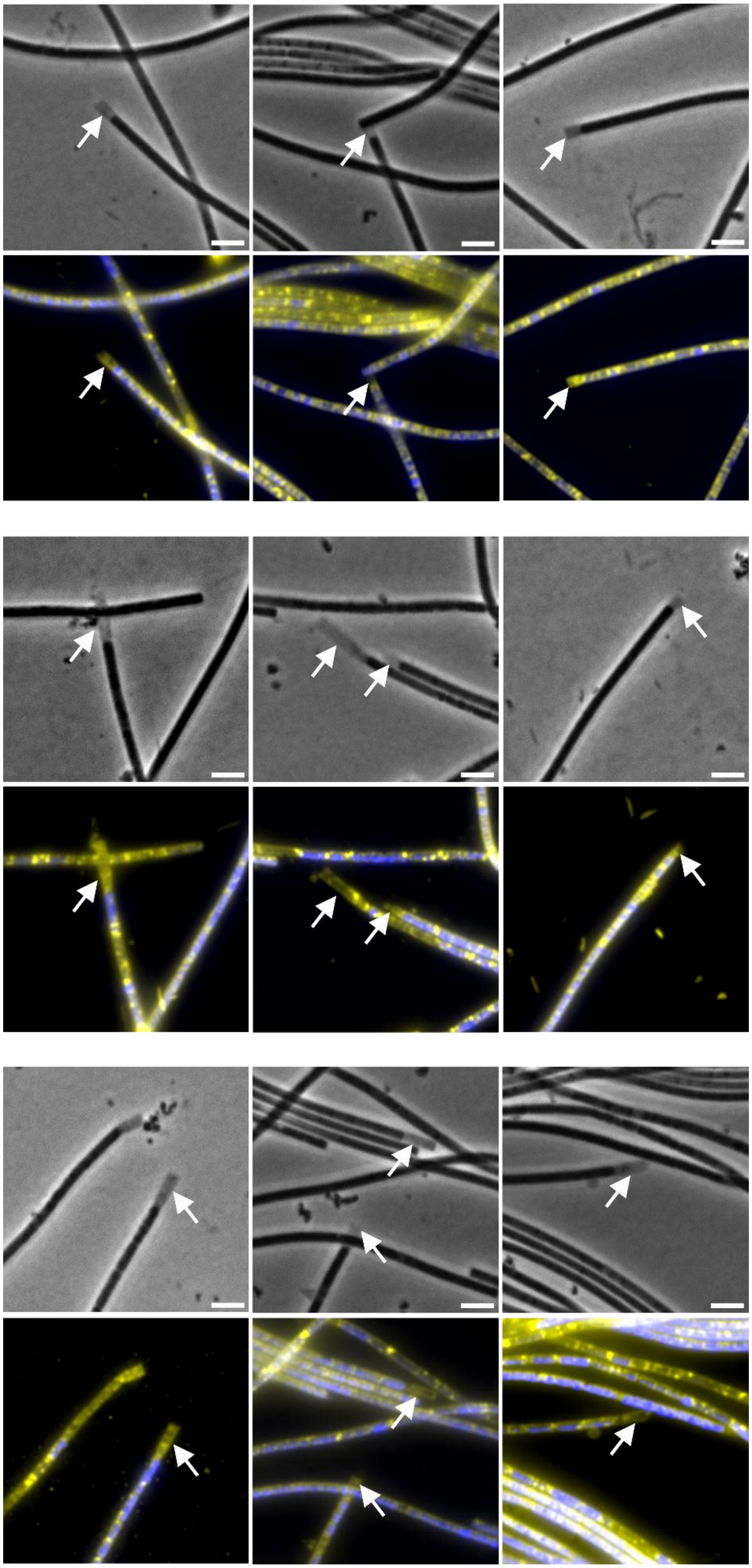

Supplementary Figure 1. Light microscopy images of *L. aerophila* cell. Representative light microscopy images of *L. aerophila* cells exhibiting polar segments with no DNA signal (white arrows). Upper panels show phase-contrast images, the lower panels show the corresponding fluorescent images. Membranes were stained with Nile red (yellow), DNA was stained with Syto13 (blue). Scale Bars 3 µm.

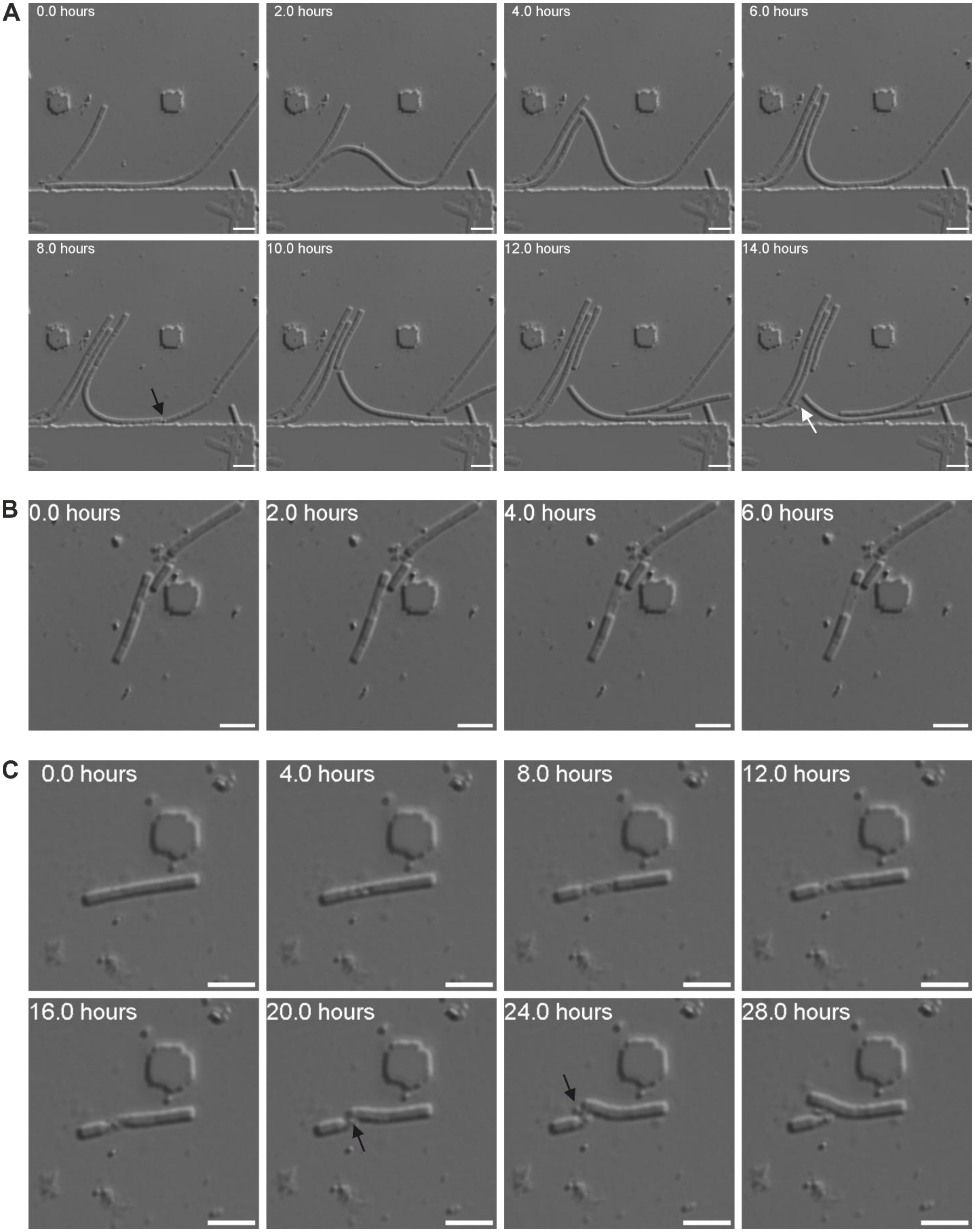
A
0.0 hours
2.0 hours
4.0 hours
6.0 hours
8.0 hours
10.0 hours
12.0 hours
14.0 hours
B
0.0 hours
2.0 hours
4.0 hours
6.0 hours
C
0.0 hours
4.0 hours
8.0 hours
12.0 hours
16.0 hours
20.0 hours
24.0 hours
28.0 hours

Supplementary Figure 2. Time-lapse microscopy of *L. aerophila* cell division. Three representative time-lapse series show sequential stages of cell breakage resulting in shortening of the multicellular filaments. Time stamps indicate hours post-imaging initiation.

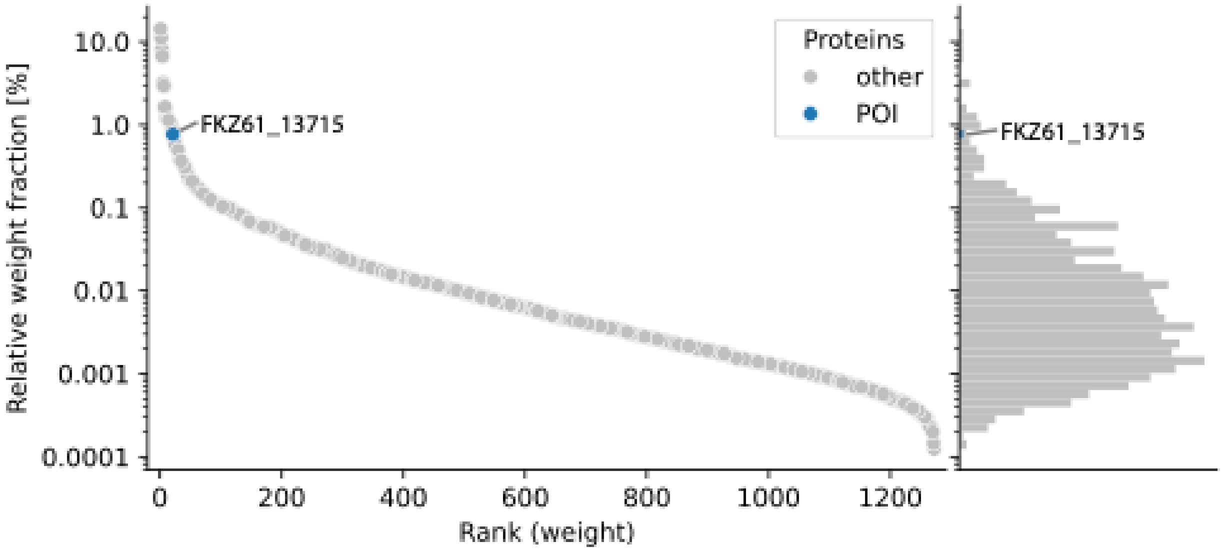

Supplementary Figure 3. Quantitative MS analysis of CsCl gradient fractions of sheared filaments from L. aerophila. Ranking of all proteins by their relative weight fraction based on intensity-based absolute quantification (iBAQ) places WP_141610714.1 as 21st.

Supplementary Table 1. Tad-Pilus encoding genes from the *L. aerophila* genome. The table contains an overview of the found proteins, accession numbers, residue count, an annotated description and possible function.

Protein	Accession Nr.	Residues	Annotated Description	Possible Function
**Flp/PilA**	MCC9078015.1	66	Flp family type IVb pilin	Major pilin
**TadE/CpaJ 1**	MCC9078014.1	110	Pilus assembly protein	Minor pilin
**TadE/CpaJ 2**	MCC9078016.1	148	Pilus assembly protein	Minor pilin
**TadZ/CpaE 1**	MCC9078012.1	386	Response regulator	ParA/MinD ATPase
**TadZ/CpaE 2**	MCC9078019.1	407	AAA family ATPase	ParA/MinD ATPase
**RcpC/CpaB**	MCC9078018.1	267	Flp pilus assembly protein CpaB	Inner membrane subunit
**TadB/CpaG**	MCC9078021.1	311	Type II secretion system F family protein	Platform protein
**TadC/CpaH**	MCC9078022.1	310	Type II secretion system F family protein	Platform protein
**TadA/CpaF**	MCC9078024.1	499	CpaF family protein	Motor ATPase
